# Foreign Medico-Psychological Literature

**Published:** 1862-01

**Authors:** 


### FOREIGN MEDICO-PSYCHOLOGICAL LITERATURE.

OUR Retrospect of current Medico-Psychological Literature will embrace the following subjects :

1. On Lesion of Association of Ideas.
2. On the Organization of a Lunatic Asylum.

### 1.—*On Lesion of Association of Ideas.*—By M. LE DR. BILLOD, Chief Medical Director of the Asylum of St. Gemmes.

THE following is a translation of a paper read before the Medico-Psychological Society of Paris, and which the author tells us forms a chapter of a work on pathological psychology, which he hopes some day to give to the world:—

"The human intelligence does not alone acquire ideas and submit them to certain transformations; it can also in the absence of the objects which have furnished these ideas, recal them even though they may have been forgotten for a time, and the manner in which they are recalled demonstrates the existence of a faculty, whose function is to seize certain relations between ideas at the moment of their acquisition and to associate them together in consequence of these relations. When the memory operates it is observed that the idea recalled has a certain relation, a certain resemblance to an idea which occupied the mind previously, and it is on account of this *rapport* that we pass from the idea which occupied us, to that which has been recalled to our memory. Our ideas, by reason only that we can recal them, are so connected one with another, that none are properly speaking isolated in our intelligence, but they are enchained and bound together in such a manner that if one of them reappears it is followed by others, which have again certain relations of their own, from which it clearly results that our minds have, at the moment of perception, the power of forming these trains, these connexions of ideas which sustain and suggest one another, and this is the power which in psychology is called the association of ideas:

"A great number of facts find their explanation in this faculty. Such as dreams and reveries, the succession of ideas on which depend conversation, digression, &c. Although the association of ideas is exercised involuntarily, it is subject to certain rules. It is thus that we associate our ideas:

"1st. With relation to the recurrence of times and places; for example, when two events have transpired at the same time, the idea of one recals the idea of the other, or when we have seen two persons in the same place, the idea of one equally recals the idea of the other.

"2nd. With relation to resemblance; if we know one lame person we are reminded of another individual suffering from the same infirmity.

"3rd. With relation to opposition; thus we associate the ideas of virtue and vice, of peace and war, of pleasure and pain.

"4th. With regard to the relation between the sign and the thing signified; thus, a single word or the sight of a single object can, under certain circumstances move us powerfully even to tears by the connexion of the ideas recalled to our minds with those expressed by the word or the object.

"In fine, we associate our ideas in an infinite number of ways, which would be tedious to enumerate. It shall suffice to establish a distinction between them which appears to us to be fundamental.

"Some are seized instantaneously without trouble or effort by the mind. The relations we have first enumerated are of this kind, and the associations derived from them are called *accidental associations.*

"Others, on the contrary, must be realized by a certain effort, a certain application, and neither their existence nor their developmeut depend on chance, nor is, as in the preceding cases the result of accident. These relations have, therefore, received the name of *constant relations;* such are the relations of cause and effect, of means to the end, of premisses to conclusions. The associations founded on these relations are called systematic or philosophic associations.

"The habit of associating the ideas in accordance with accidental or with constant relations causes a marked difference between different orders of mind. Those who naturally or by force of habit seize only accidental associations, see only the pleasing and superficial aspect of things. Such are in general wits, poets, and men of imagination—all those who show a particular aptitude for versifying, for making ingenious comparisons, for metaphors, for spiritual allusions, for sallies of wit, and play on words; and those who seize only constant relations, and make in consequence only systematic associations, form the class of thinkers, of *savants,* and philosophers, and, while the former shine by their wit and imagination, these last are distinguished by their judgment.

"Among the effects which result to the intelligence from the exercise of this faculty, is one on which we must at present insist, for its influence is especially discoverable in cases of insanity; I allude to the false judgments which are caused by a vicious association of ideas, and which almost always result in a tendency to substitute a constant for an accidental association. Among these false judgments may be mentioned the popular superstition connected with the number 13, with the overturning of salt-cellars, with certain lunar influences, the *post hoc ergo propter hoc* in medicine, and all the errors which follow when the mind takes a relation of coincidence for a relation of cause and effect, and thus substitutes a constant association of ideas for one which is merely accidental.

"Applying the preceding propositions to the study of mental alienation, it would be easy to demonstrate the influence exercised by the associations of ideas upon the manifestations of delirium. In the general delirium of acute mania, this faculty participates in the general excitement, and the rapid succession of the associations of ideas is the result. Let us add even that the rapidity of this succession is such, that utterance failing to give expression to it, there results an incoherence which is perhaps only an incomplete translation, and must not be confounded with the incoherence of demented persons, which is caused by forgetting certain words. Besides this, the confusion in the associations of ideas is general, and in the *pêle-mêle* which follows, it is no longer possible to distinguish between the associations of accidental and of philosophic ideas, although the former would be expected to predominate. The habit of rhyming practised by certain maniacs in their delirium is explained by an exaltation of this faculty.

"Now if we pass from general delirium to partial delirium, it will be easy for us to illustrate by cases the influence of disturbances in the different manifestations of the association of ideas, and to show by example that the error of judgment in which delirium consists, results frequently from the fact that the lunatic mistakes a relation of coincidence for a relation of cause and effect. Let us review in this connexion the different forms of partial delirium, and commence by hypochondriacal delirium. Is it not evident that the hypochondriac substitutes a constant for an accidental association of ideas, when he mistakes the symptoms of a cold for the symptoms of phthisis, or if suffering from gastralgia thinks he has schirrus of the stomach? He founds in this case a constant association of ideas on a relation of resemblance which is only accidental. It is by a vicious association of ideas, and by mistaking a relation of simple coincidence for a relation of causes and effect, that some insane persons have persisted in refusing food, the eating of which has coincided with certain sufferings, though they could not possibly have been occasioned by it.

"To a vicious association of ideas may be referred the following aberrations, analogous instances to which are constantly occurring in the practice of alienists.

"One of my patients refuses to wear a dress of the colour of chocolate, because every time she wears it her digestion is affected in the same way as if she had been taking chocolate. This same invalid fancies she cannot sleep in a room with a parquetrie floor when her bed is placed in a certain position.

A lunatic of the Asylum of Blois, who performed in my time the duties of secretary there, attributed influences fatal to his health to the colour of blue, and to neutralize these influences, whenever he wore a garment of this colour he placed in his buttonhole a morsel of green cloth or the leaf of a tree, green being, as he thought, beneficial. Blue, he said, gave him the colic. He attributed the expulsion of some worms one day to having been in a place where was some glass surrounded by a kind of frame. He thought he had lost his hair and part of his beard after a journey in Sologne, and in consequence of the aridity of the soil in that country.

"Certain figures, the figure 7 for example, were subject to malignant influences: he refused to work the 7th of each month, because he had been confined by the influence of a family consisting of 7 members, who had obliged him to work with a pickaxe, the form of which resembled No. 7; and he accounted one day for the uncertainty of his handwriting and the want of straightness in the lines, by the visit of a lame physician to the asylum.

"One of my inmates at St. Gemmes, who was a priest, constantly attributed certain influences on the weather to the effect of his prayers. Certain gestures of this or that person made him sneeze or blow his nose, announcing to him some sort of modification in his health, which the invalid described, saying they had given him '*l'antienne.*' This same ecclesiastic, believing that the reigning sovereign of France was named Henry V., King of France and Algeria, when the sky was covered with clouds during the singing of the *Domine salvum fac imperatorem* in the chapel, attributed it to the influence of this chant. Another priest, now quite recovered, supposed in his delirium that all the ills which afflict humanity were caused by the eating of apples, and consequently abstained from this fruit himself, and wished that all the bishops should issue a proclamation commanding the destruction of apple-trees. Such an aberration results evidently from a vicious association of ideas of a mistaken relation between the particular use of the forbidden fruit on which the dogma of original sin is founded, and the use of apples in general. Another of my patients held in his hand one day the fruit of the *Datura stramonium*, or thorn-apple, which he had gathered while walking in the country. When I requested him not to touch it lest it should poison him, he replied, 'Oh, when these fruits are tinned over there is no danger.' A mistaken resemblance between the copper vessels which are tinned inside to prevent their injurious effects, and the poisonous plant which he held in his hands.

"An inmate at St. Gemmes, merely because he had suffered from a nervous attack after something had been offered to him with the phrase, '*Voulez-vous?*' could never hear these two words without urgently requesting that I would order the person never to use them again in speaking to him.

"The voices which many persons suffering under hallucinations fancy they hear frequently pronounce sentences in rhyme.

"One of the lunatics in the asylum at Blois was continually writing on a slate a sentence expressing always an association of ideas, and adding to it afterwards the letters composing the phrase. The total revealed to his recollection the date of one of the important events of his life—such as his birth, his marriage, the death of his wife. He expressed this relation in a sentence which he wrote on the other side of his slate, of which he added also the letters, and so on. He wrote one day *voltigeur* and *soleil.* 'Why do you associate these two words?' I asked.—'*Parceque les épaulettes des voltigeurs sont jaunes, et que le soleil l'est aussi.*' These two words being formed of fifteen letters, this number recalled to him an event which occurred in the year 1815.

"While the manifestations of hypochondriacal delirium are influenced by certain associations of ideas, the facts which we have just cited would prove that the manifestations of other forms of melancholy delirium have also the same issue. We will try to prove this by some special examples.

"By finding himself in the same place with certain persons, one of my patients supposed that he was threatened with the greatest misfortunes. Another believed that if one accosted him on the left side instead of the right, some evil would happen to him, and if he were so accosted, he hastened to neutralize the injury by a countermarch.

"One inmate of the Asylum of St. Gemmes, believed himself to be damned from the time of wearing some braces given him by a woman who had bewitched him. We may add that the aberration of ideas which consists in believing that one has been enchanted or the victim of persecutions, proceeds from a vicious association of ideas.

"One of my insane patients who supposes that she is being burned by some one at a distance, asserts that the place she inhabits is not St. Gemmes, but St. Brûlant.

"The construction put on all that is said and done around certain patients suffering from melancholia, the hostile intentions which they ascribe to every one, are not these the result of the erroneous association of certain facts with others which have no connexion with them? Let a lype-maniac imagine, for instance, that any one who spits in passing near him, does it with intention to insult him, it is evident that this error in judgment is caused by an erroneous connexion between an action purely fortuitous and an intention which only exists in the mind of the lunatic.

"It is only by a lesion of the association of ideas that a fact can be explained which we have never seen mentioned anywhere, but which we have in two cases observed ourselves: that certain persons affected with a delusion on the subject of persecutions, fancy they have a *sosie* (double ?), imagining, for example, that this double, which, in the case of one of my patients was the singer Rousseau-Lagrave, was subjected to precisely the same persecutions as themselves.

"We believe that all the predominance of ideas in a fit of delirium, results from an association of ideas to some extent irresistible and founded on forced relations. Thus it is in religious delirium, for example; the association of ideas is founded on relations of semblance with regard to religious subjects; in hypochondriacal delirium it rests on the relations of resemblance with regard to the sentiment of personality.

"We have already published in the *Archives Cliniques* of M. Baillarger, and we think it desirable to reproduce it here, a rare, not to say unique example of association of ideas with regard to relations of resemblance both geographical and historical.

"M. D. . . Pierre (de Saumur), *ex greffier de justice de paix*, aged 63 years, entered at the asylum of St. Gemmes the 21st May, 1850, after a previous confinement of more than 20 years at the hospice *de la Providence* of Saumur, is a man of sanguine temperament, and of vigorous constitution, although of short stature. His physiognomy is open, intelligent, and stamped with an expression both of good nature and of cunning.

"His mental alienation commenced more than 33 years previously, and exhibited at first marked characteristics of mania with general delirium, and a tendency to exaltation and fury.

"After this exaltation had lasted some time it subsided, and left the invalid in a peaceable state of mind, except when suffering from physical discomfort, he exhibited occasionally a slight irritability. As to his delirium, it was finally characterized by a predominance of geographical and historical ideas, the incoherency of which it is impossible to describe.

"To give a correct description of the mental state of M. D——, I cannot do better than let him speak for himself, and give a sort of short-hand sketch of some of his conversations taken at random.

"The 5th of February, M. D—— to the question, 'How do you do?' answered:

"'There are seven communes of the environs of Jersey, for the commune of Villebernier, and that is why the oxen of Polish Saxony arrive for the diploma of the Eastern Pyrenees. It is by order of the town of Forcester, and because of the assistance of two marshy rivers of the Saturnine Olympiad, which Olympiad is fixed in the sign of cancer of the French Republic; the Jaspy sheep arrive this morning.'

"July 7th, he replied to the same question:

"'The Polish Algarves are in good condition. Le Nivernais is plentiful in oxen, and the dedication of the stump for Alexis Premont is finished; he acts with two bushels of earth to the body planted with chesnut trees; this is an auxiliary appanage of the second Sapinière de la Palance, on account of the town of Dauvres, capital Stamboul.'

"Feb. 12, asking him whether he had any news, I received this answer:

"'To-day, sir, there is nothing. There is only Saintonge and the country of the Nograis Tartars for the assassination of the Duke of Berri. That comes from Novogorod, and from Varsovie, and from Du Meurain, and from the walls of China, and from the country of the Carloman Tartars, and from the territorial divisions of Great Varsovie, which they call the Episcopal destinies, the sea-coast of St. Salvador. It is for the reception of the young Menuise at the Hospital of the Fifteen-Twenties, for Saxon Bavaria, Suabia, Poland, the plains of Cairo. They call that the reception of Carlori, Saxon Bavaria for the episodes of Nuremberg to the groves of Puytrol, where it formed the second time the Holy Hermandad for Moldavia; there is the source of the apostolic junta which has been formed for the Cracovian Turk meridian, which carried away the Arabesque Condom in 1801 and 1802 for the merchants of the arabesque village, where is at present held the fête of the Dutch Panegyric for the fortress of Baden, of Utrecht, of Amsterdam, and of Roberta.'

"To these quotations, which completely picture the invalid, and which might be endlessly multiplied, we must add some specimens of his poetical lucubrations:—

"'Viens, viens, mon très cher Eugène,
Viens, viens, revoir ta carène,
L'Indoste suit toujours Tamerlan;
Tu prends le casque de l'éperlan;
Tu vas renaître sur le mont Acide
On y place l'étendard d'Alcide.
Tu porteras chez nous la sainte dague,
Tu verras les clochers de Copenhague.

"'La belle Amidonne a ses beaux jours;
Il reste pour toi quelques amours;
Elle est princesse de la rivière,
Elle a les présents de la tourière,
Près de la colonne de Trajan.
Tu vois aspirer la fin de l'an;
Réjouis-toi, les souvenirs d'Auxonne
T'assurent une frêle couronne.

" 'Voici l'étendard de Gengiskan,

Qui le porte vers le Ramadan;

Il vient des plaines de la Salène

Relever la martre qui nous mène.

Reçois l'hommage de notre foi,

Va, reste au principe de la loi;

Un jour tu verras la Victorine,

Ses armes, sa robe purpurine.

" 'Je suis un bon tire-ligne,
Je vais aux mers du Japon,
J'aime la pêche à l'esprigne,
Je suis le jeune Alcyon,
Quand je brave la froidure,
De Neptune j'ai le trident,
La douce température
Me retient à l'Océan.

" 'Après un bal tout champêtre,
Encase le casoard,
Sortant de l'ombre d'un hêtre
Me nomme le Balthazard.
Je pars pour la couleuvrine
Sondant le fond du torrent,
Et quand le chagrin me mine,
On vient m'arracher une dent.

" 'Après avoir rendu des services
A l'État si florissant,
D'une belle j'ai les prémices,
Et je deviens le géant
De la rue Coquillère;
Je tourne aussi les fuseaux,
Je porte la genouillère
Avec l'enclume et les marteaux.' "

LA TARENTULE.

Une tarentule en une circonférence
D'un pouvoir invisible admirait la puissance,
Et par sa fragilité se tenant sur l'eau,
Éblouissait nos yeux par un jeu de cerceaux.
C'est bien une merveille et je puis vous le dire,
L'observateur curieux y jette son sourire.
Monsieur de Buffon par un élan studieux,
Montre aux Astèques le miracle de cieux.
Et des archers du fisc, l'histoire naturelle,
Amis, nous en offre la relation ardelle.

" After having dictated these last verses, M. D—— said to us, 'Here are a hundred little verses, sir. They are dressed in uniform in greyish blue, with little buttons of pewter. They come from Lozerè.' We must add, that when a book was opened before M. D——, in which were some lines of writing, he substituted, in the place of the text, phrases of his own, of the same kind as the specimens we have given. Thus, of several addresses of letters presented to him, he read on the first, ' For the garrison of Charente, for the house Giron;' on the second, 'For the gilded well of St. Nicholas, for the inhabitants of la Gironde;' on the third, ' For the rabbis of the Eastern Pyrenées;' and on the fourth, 'M., the sedentary officer of Mont Cenis, for the *officiers perrayeurs de St. Germain.*'

" It is difficult to fix the attention of this lunatic; but with a little perseverance that can be sometimes accomplished, and it is then possible to obtain from him direct and sensible answers, which show that his memory remains perfectly unimpaired.

" If the exaltation or the deviation of the association of ideas forms one of the essential characters of delirium in all forms of mental alienation, it may be affirmed that its enfeeblement or its destruction is one of the essential characters of dementia.

" Dementia, as is well known, breaks the chain of the ideas.

" The mind of a demented person ends by being insensible to any relation, and the incoherency of ideas is of course complete. As to the rest, in dementia, as in all the other phases of mental alienation, the lesions of the associations of ideas connect themselves with those of the memory, as we shall show when we treat of lesions of this latter faculty."

---

2.—*On the Organization of a Lunatic Asylum.* By Dr. E. Renaudin.

The following Report on the Organization of a Lunatic Asylum has been recently prepared, for the information of the Prefect of the Department of the Seine, by Dr. Renaudin:—

"The data of this organization are connected with two orders of facts.

"The one, nearly independent of the effective strength of the population, corresponds with the fundamental bases of the service defined by the laws and regulations.

"The others, on the contrary, determine the individual government of each inmate of the asylum.

"The laws, ordinances, and decrees establish formally the administrative autonomy of the asylum, which should possess the advantages of civil life, and which has all the characters of a foundation of public utility, whether the expense of its construction has been defrayed by public taxation, or private bounty has added to its endowment. The law has determined the bases of the assistance, fixed the concurrence of the department and the communes, and regulated the constituent elements of the tariff which should meet all contingencies, without the department being ever called upon to provide a subsidy beyond the amount of the board which it pays for its invalids, and after deducting their time of abode there (apres décompte leurs journées de présence).

"In virtue of this fundamental principle, the asylum has a special administration: its private funds, its own resources, its private budget, and hospital legislation regulate all the details of its management.

"Masters of the science have always agreed on laying down the principle that the administration of an asylum should be essentially medical; and that, consequently, a physician must be the sole governor of that colony of which the members, become foreign bodies in society, are called to constitute a society *sui generis*, into which each brings the contingent of his previous aptitudes (antecedent dispositions). It is the medical judgment which directs these dispositions; and from the moment when the physician confines the potentiality of direction to himself, he cannot fulfil his high mission except in the condition of being the real head of the medico-administrative service.

"To place an institution of this kind under non-medical government would, in my opinion, constitute an anomaly as striking as the appointment of a priest to the command of a regiment, or of a colonel to the tutorship of a seminary.

"In Germany and Italy this truth has long been an axiom. And if, in France, general inspection has given to the service the impulse of which we now prove the excellent results, it is because it has been, and still is, entrusted to eminent alienists, who have *medicalised* administrative science, and have adapted it to the numerous indications of psycho-curative knowledge. Physicians are substituted for the charitable but uninstructed administration of religious communities, and everywhere the direction of the insane has beneficially experienced this important reformation.

"If management has been improved by the breath of science, the latter, in its turn, has become much more practical by its connexion with administrative forms. In place of being misled by vague theories, the administrative physician (medical director) is in closer proximity to his patients, knows them better, appreciates their wants and forms his deductions from facts well observed rather than from preconceived theories, too often falsified by experience. A mass of arguments demonstrate the truth of this assertion, for it is from medical directors that the most practical and most important works have emanated. Their position has admitted of their separating the romance of the disease from its history. It is they who have best delineated insanity by stripping it of its stage-dress; and if their works have not had much notoriety, it is because these practitioners, exclusively devoted to their duties, are less concerned for the care of their reputation than for the welfare of the invalids who are entrusted to them.

"These remarks already foreshow how far the medical service of asylums must differ from that of ordinary hospitals. In the latter, it is a simple episode incident to the existence of the physician; in the former, on the contrary, it absorbs the whole life of the practitioner, who cannot know his patients except by dwelling among them, and whose habitual residence in the establishment is an essential element of that moral hygiene so important at the present day. In like conditions, the administrative functions, instead of being an additional burden, are, on the contrary, a powerful auxiliary of the treatment, if the organization correspond with the fixed requirements of the duty.

"The selection of the steward and the housekeeper contributes to lighten the task of the medical director. If these are respectable and able functionaries, the supervision of their duties is simplified. In short, the administrative functions acquire all their moral worth if the director is effectively seconded by a head clerk capable of keeping properly all the books connected with the establishment and the general accounts.

"On the other hand, the numerous details of the medical service require that the director should have an assistant physician and house-surgeons (internes). An apothecary ought also to be attached to the establishment.

"As a plan of organization cannot be judged in its connexion with the whole except by giving to it a numerical expression, I have taken for a basis the salaries allowed in the provinces, increasing them by one-third, as is practised in the payment of troops when quartered in Paris.

| | | |
|---|---|---|
| "1 Medical Director . . . . . | 8000 | francs. |
| 1 Receiver acting as Steward . . . | 6400 | " |
| Carried forward, | 14,400 | " |

| | | |
|---|---|---|
| | Brought forward, | 14,400 francs. |
| 1 Assistant Physician | . | 3200 „ |
| 2 House Surgeons (or clerks) | . | 1600 „ |
| 1 Apothecary | . | 2400 „ |
| 1 Principal Clerk of Management | . | 2000 „ |
| 1 Entering Clerk (Bookkeeper) | . | 1500 „ |
| 1 Copying Clerk | . | 1200 „ |
| 1 Accounting Clerk in Steward's Office | . | 1800 „ |
| 1 Clerk of Distribution | . | 1200 „ |
| 1 Storekeeper | . | 1200 „ |
| 1 Almoner (chaplain) | . | 2400 „ |
| | Total, | 32,900 „ |

"All should reside in the asylum, and then will be entitled to the allowances of firing and lighting, which approximately will make up, including therein the food of the House-Surgeons, valued at 1000 francs, a total expenditure of 4100 francs.

"This general expenditure will then be 37,000 francs.

"The officers of the different general duties form a second class, composed of the following elements:—

| | | |
|---|---|---|
| "1 Door-porter | . | 600 francs. |
| 1 Assistant Door-porter | . | 400 „ |
| 2 Gardeners | . | 1400 „ |
| 1 Head Cook | . | 800 „ |
| 2 Assistant Cooks | . | 800 „ |
| 1 Baker | . | 600 „ |
| 1 Butcher | . | 600 „ |
| 1 Chief Sempstress | . | 500 „ |
| 3 Needlewomen | . | 900 „ |
| 5 Head Workmen | . | 3000 „ |
| | Total, | 9600 „ |

"These eighteen officers, fed, clothed, and their washing done in the asylum, cause the following outlay:—

| | | |
|---|---|---|
| "Food, at the rate of 365 francs each | . | 6570 francs. |
| Clothing | . | 1800 „ |
| Washing | . | 180 „ |
| | Total, | 8550 „ |

"If the staff of persons employed in the direct supervision of the invalids may, in certain conditions, depend on that of the invalids, the methodical classification necessary to be adopted for them constitutes the first indication of a normal framework. In general eight sections are admitted in each division. The individuals of each division are under the orders of a head, who centralizes the service. In the division of the men there must, therefore, be enumerated a chief superintendent, twenty-four officers, and a bath-man. The like number, of their own sex, are required for the female division.

"Here a question, which has been raised at various times, presents itself for examination. Is it beneficial that the asylum should be prepared for the reception of both sexes, or would it be better that it should contain only one? Experience has shown that the reception of the two sexes presents indisputable advantages in an economical point of view. There are, so to speak, two asylums in juxtaposition, which lend to each other a mutual support, and discipline is maintained with equal ease if the local arrangements ensure the complete separation of the sexes.

"The good order of an asylum depends especially upon the proper selection of the officials charged with immediate attendance on the invalids. There are many asylums in which a lamentable parsimony is opposed to a good organization, and opens the door only to incapable persons. A good staff of officials, and adequate accommodation, are the principal elements of a moralizing discipline. According to these principles the expenditure should be made up thus:—

| | | |
|---|---|---|
| "1 Principal Superintendent . . . | 1800 | francs. |
| 4 Superintendents . . . . | 2400 | " |
| 6 Under-Superintendents . . . | 3000 | " |
| 8 Nurses of the 1st class . . . | 3200 | " |
| 6 Nurses of the 2nd class . . . | 1800 | " |
| Total, | 12,200 | " |

| | | |
|---|---|---|
| "1 Female Principal Superintendent . | 1200 | " |
| 4 Female Superintendents . . . | 2000 | " |
| 6 Female Under-Superintendents . . | 2400 | " |
| 8 Female Nurses of the 1st class . . | 2400 | " |
| 6 Female Nurses of the 2nd class . . | 1200 | " |
| Total, | 9200 | " |

"The principal Male and Female Superintendent wear the costume adopted for the asylum; but the value of their support is included in their salaries by reason of their being connected with the superannuation fund. As for the other forty-eight officials, their food represents 17,520 francs, their clothing will cost 4800 francs, and the other expenses amount to 480 francs; making a total of 22,800 francs.

"In making a summary of the preceding details, we see that the whole expense of the staff represents a sum of 99,350 francs.

"Taking this expenditure at a net cost of 25 centimes, there would require an effective force of 1080 patients.

"Admitting a value of 30 centimes, the number would then be 900.

"An average of 40 centimes corresponds to the figure of 675. This is the effective at which you are stopped, and it is also that which corresponds with the principal indications. It gives to each section an effective mean of 42 invalids, and causes no necessity for augmenting the superintendence, which, in the other hypothesis, would have burdened the individual expenses.

"The general expenses will, therefore, include the following elements:—

| | |
|---|---|
| "General expenses: personal | 0·40 |
| " " material | 0·02 |
| Repair of buildings and furniture | 0·05 |
| Lighting | 0·03 |
| Firing | 0·04 |
| Total, | 0·54 |

"This is only one centime more than at the asylum of Auxerre, which, by its position, may allow lower salaries than at Paris, where the salaries are much larger.

"In the preceding considerations, I have supposed an exclusively lay service, such as exists at the asylum of Auxerre. The admission of a community of nuns in the female division would occasion an expense much greater in respect of food than that of number. This increase cannot be valued at less than 4000 francs, or at about two centimes *per diem.*

"We have already seen that in the prevision of the effective force which we have admitted, each section was composed of a mean of 42 invalids. The buildings being constructed on an average of 54 locations per ward, we may presume that the population of the asylum may, without disturbing the economy of the organization, undergo some eventual augmentations, which, at the rate of five invalids each section, would constitute a total fluctuation of eighty. Beyond that term, the limits of superintendence would be modified; and if the part of the fixed number was considered as a less proportion in the value of the net cost, that alteration would be compensated by the increase of the number of superintendents. In other words, the alteration of the general expenses has a limit, and it is a term beyond which the increase of the effective force no more presents any financial advantages.

"In order to avoid all misapprehension, I have calculated the general expenses widely; but the fluctuations of the value of commodities, the vacancies of office, the variations of the effective, the conditions of advancement in every grade, sometimes effect partial reductions, which allow of this programme being estimated as a maximum.

"After this rough estimate of the general expenses of organization, the estimate of the personal cost of each lunatic naturally suggests itself. I cannot here, sir, enter into all the details of a mass of observations; it will suffice to place before you the general results, to show the influence on the value of the net cost.

"We must first consider the portion of individual expenditure apart from the alimentary system.

"These are:—

| | |
|---|---|
| "Washing for | 0·025 |
| Bedding for | 0·025 |
| Linen and clothing for | 0·085 |
| Medicine for | 0·015 |
| Tobacco for | 0·010 |
| Total, | 0·160 |

"With respect to food, the expense is divisible in two parts: the one relative to the preparation properly so called represents nearly a value of two centimes; the other comprehends the elements properly so called, and their condimentation. In relation to this second part, we observe that annually each invalid must have 255 kilogrammes* of bread, 70 kilogrammes of animal food, and 85 litres† of wine; which constitutes for these three articles an annual outlay of about 181 francs 60 centimes. As for the other provisions and articles of condimentation, we cannot calculate them at less than 20 centimes *per diem*, or 73 francs *per annum*. The whole expense of food then would be about 255 francs, or 0·69 *per diem*.

"According to these observations, the daily cost for individual expenditure would be 0·85.

"The total daily cost, comprising therein the general expenses, would therefore amount to 1 franc 39 centimes; and the yearly cost of a lunatic would be 507 francs 35 centimes.

"This result shows at first that boarders admitted to the common system (diet?) must be at the rate of 1 franc 45 centimes *per diem*, in order that the asylum may never be exposed to suffer the fluctuation in price of commodities.

"Boarders at 2 francs *per diem* might enjoy a certain amelioration of *régime* on condition of being, in a separate section, subjected to the common mode of living.

"To have a private apartment and more varied diet, the boarders should pay 4 francs *per diem*.

"And, lastly, if special attendance be united to private apartment, a daily charge of 2 francs 75 centimes must be added to the principal cost of the preceding class.

"These indications suffice to show the utility of the boarding system in a public asylum of which the financial position improves itself, at the same time that families find in it, at less cost, attentions and legal guarantees which they might in vain look for elsewhere on the same terms. In like manner, as regards the poor, the value of the net cost is here a question of number; but at the commencement of an organization, it would be imprudent to calculate *à priori* the portion of profit to be gained upon an uncertain receipt.

"Thus far, sir, I have looked at the asylum as exclusively a consumer. It is now necessary that I should exhibit it as an intelligent and lucrative producer.

"In this production two essential elements concur—the labour of the lunatics, and the implements of that labour.

"In comprising head workmen in the number of officials, I have already pointed out that, for the sake of treatment and discipline, the internal life of the asylum should reflect what goes on outside its walls. At Paris especially, the industrial element ought to play an important part in the existence of the establishment. Therefore, in calculating certain expenses, I have taken into account the organization of the

* Kilogramme = 2 lb. av.

† Litre, somewhat more than 2 pints Eng. (= nearly 2·1135 pints).

work. The repair of the furniture and building obtains valuable assistance from the workmen of every trade. The cost of linen and clothing, as represented in my calculation, only refers to the purchase of the new materials, as all the articles ought to be made in the asylum, and some saving might also be realized by the introduction of looms. The working element is accordingly of great importance; and it has been already considered in my calculations, which otherwise would have been much higher if we had to depend upon out-of-door industry. The various details of internal service obtain much also from the co-operation of the insane. The washing and cleaning find valuable assistants in the lunatics, without whom the proportion of officials would require to be augmented. This part of the labour has, therefore, already paid a large tribute, since it has reduced the value of the net cost and is not accounted in the tariff of daily charges. The other element of production is produced by the agricultural labour, which constitutes a truly effective receipt. This receipt is of a mixed character, and its valuation comprehends in one part the interest of the piece of ground, and in the other the work by means of which this ground produces the articles of consumption. These products are divisible in two classes. The one, industrial, resolve themselves into other products, sowing for cultivation and consumption: such are fodder and plants fit for fodder, as well as manure; others, on the contrary, enter directly into the general consumption. This distinction, it appears to me, ought to be taken into very serious consideration in determining the topographical situation of the establishment. If it should be situated in the centre of a large piece of ground, there is opportunity for placing on the side of the females meadows, natural and artificial, as well as plants fit for fodder; whilst on that of the men might be laid out the portion intended for kitchen-gardening, which is the most productive in an asylum, because it requires more active manual labour and less expensive materials. To complete these various indications, the asylum ought to be able to cultivate twenty hectares, whereof ten should be set apart for the pasture of animals and ordinary cultivation; whilst the labour of the insane would convert the other ten into a kitchen-garden, providing for internal consumption products which they could not procure for themselves in common. Without entering, sir, into the details of the elements of this cultivation, I may give you an idea of its approximate results:—

| | | |
|---|---|---|
| "Sixteen cows will produce a value of | 6,000 | francs. |
| "Fifty hogs will give . . . . . | 5,000 | " |
| "The produce of the garden may probably be valued at . . . . | 24,000 | " |
| "Total . . . . . . . | 35,000 | " |

"From this must be deducted—

"1. Gratuities to the labourers, 4000fr.; 2. The cost of materials of cultivation, 4000 francs,—8000 francs, which reduces the net product to 27,000 francs.

"In this case the internal (domestic) production will reduce the cost of the day by 10 centimes; and the asylum established on the conditions I have had the honour to lay before you, might operate in returning to the department the cost of 1 franc 35 centimes, 5 centimes being set aside to form the sinking fund of the contingencies to which the fluctuation of the prices of certain commodities exposes it.

"The casual receipts of different kinds, the profit realized upon the boarders, would form for the asylum a habitual excess of receipts, the employment of which might be determined after fixing the quota of a rolling fund, which, lodged in bank (the Treasury), would enable the asylum to meet all its engagements with a regularity favourable to its interests.

"Such, sir, is a summary analysis of the element of the budget of an asylum, not such as the rules of accounting prescribe for establishing it with the complication of its receipts and expenses of management, but in its results separated from the intermediate operations of transformation. To give a more obvious form to the argumentation, I have been obliged to have recourse to valuations which can be easily rectified if they are not thoroughly accurate. I have supposed that there is in the asylum a bakehouse and slaughter-house; I assume that the asylum is situated beyond the limits of city taxation; and especially I suppose that at the moment of commencing, its material organization is complete.

"As you properly observed on your visit to Auxerre, that which has made the financial statement of certain asylums very difficult, is that in general attention is only paid to the buildings, that frequently even they remain unfinished, and that moreover the care of providing painfully the furniture, napery, and clothing is left to the cost of the day. In order that the institution should work regularly, it must be established complete in all parts, with all its potentiality of action; in a word, it must be able to live the moment it sets itself up.

"It is not from the beginning that the whole of these results can be obtained. The first steps of the management will be difficult and embarrassed, its efforts will not attain at the first start to the synergy which admits of the virtuality of the institution; therefore I think that at the beginning the daily cost should be fixed at 1 franc 40 centimes. Every five years a reduction of 5 centimes might be made, until the daily cost reached the rate of 1 franc 25 centimes. At that time, the boarding department would be settled, the real foundation would have acquired the highest value of which it would be susceptible, and then it would be easy to calculate in how many years the asylum could liquidate the whole or part of its general expenses, so as only to leave to the charge of public relief the individual expense occasioned by each invalid.

"In taking into consideration the wants of the asylum, I have necessarily been obliged to lay aside the question of water. The best solution would be by building the asylum in the vicinity of a river; but if otherwise, there might arise a cost of which I cannot calculate the amount.

"P.S. The preceding considerations are applicable to an asylum constituted like those of the provinces, that is to say, embracing all the types of mental alienation. It may easily be understood that these principles would be considerably modified if it were a question of the organization of an institution embracing only the infirm, idiots, paralytics, or epileptics."

---

### A RECENT VISIT TO GHEEL.

